# Supplementary material for: Comparative Study of Single-Trait and Multi-Trait Genomic Prediction Models
Source: Animals (Basel). 2024 Oct 14;14(20):2961. doi: 10.3390/ani14202961 (PMC11506144; doi:10.3390/ani14202961)
Supplement: Supplementary file 1 [file animals-14-02961-s001.zip › Additional file 2.pdf]

Table S1. Accuracy (R2) of predicted breeding values (PBVs) by multi-trait and single-trait models at reference population sizes of 500–4500 (Scenario 1).

Reference Group Size = 500	A1B1	A2B2	A3B3	C1D1	C2D2	C3D3	E1F1	E2F2	E3F3
**Single trait model (mean/sd)**	0.118 (0.054)	0.165 (0.049)	0.238 (0.052)	0.063 (0.065)	0.127 (0.062)	0.190 (0.063)	0.105 (0.050)	0.164 (0.052)	0.231 (0.059)
**Bivariate model (mean/sd)**	0.120 (0.054)	0.170 (0.049)	0.245 (0.052)	0.064 (0.065)	0.133 (0.065)	0.199 (0.065)	0.108 (0.053)	0.173 (0.056)	0.240 (0.063)
**Improvement**	0.002	0.005	0.007	0.001	0.007	0.008	0.002	0.008	0.009

Reference Group Size = 1000	A1B1	A2B2	A3B3	C1D1	C2D2	C3D3	E1F1	E2F2	E3F3
**Single trait model (mean/sd)**	0.137 (0.047)	0.184 (0.037)	0.252 (0.038)	0.084 (0.059)	0.163 (0.054)	0.234 (0.061)	0.123 (0.063)	0.189 (0.058)	0.258 (0.066)
**Bivariate model (mean/sd)**	0.139 (0.047)	0.190 (0.037)	0.262 (0.038)	0.086 (0.061)	0.173 (0.057)	0.248 (0.065)	0.126 (0.065)	0.201 (0.061)	0.272 (0.069)
**Improvement**	0.002	0.006	0.01	0.002	0.01	0.013	0.003	0.012	0.014

Reference Group Size = 1500	A1B1	A2B2	A3B3	C1D1	C2D2	C3D3	E1F1	E2F2	E3F3
**Single trait model (mean/sd)**	0.155 (0.058)	0.200 (0.040)	0.267 (0.037)	0.100 (0.057)	0.181 (0.065)	0.250 (0.066)	0.133 (0.063)	0.203 (0.051)	0.267 (0.051)
**Bivariate model (mean/sd)**	0.157 (0.058)	0.208 (0.040)	0.279 (0.038)	0.102 (0.058)	0.193 (0.069)	0.267 (0.070)	0.136 (0.064)	0.217 (0.053)	0.285 (0.055)
**Improvement**	0.002	0.007	0.013	0.002	0.012	0.017	0.003	0.014	0.019

Reference Group Size = 2000	A1B1	A2B2	A3B3	C1D1	C2D2	C3D3	E1F1	E2F2	E3F3
**Single trait model (mean/sd)**	0.158 (0.056)	0.208 (0.040)	0.275 (0.063)	0.117 (0.065)	0.201 (0.049)	0.272 (0.048)	0.156 (0.063)	0.229 (0.052)	0.294 (0.050)
**Bivariate model (mean/sd)**	0.160 (0.057)	0.216 (0.041)	0.289 (0.070)	0.120 (0.066)	0.216 (0.052)	0.294 (0.051)	0.159 (0.064)	0.245 (0.053)	0.317 (0.053)
**Improvement**	0.002	0.008	0.015	0.002	0.014	0.021	0.003	0.017	0.024

Reference Group Size = 2500	A1B1	A2B2	A3B3	C1D1	C2D2	C3D3	E1F1	E2F2	E3F3
**Single trait model**	0.166	0.214	0.276	0.120	0.205	0.275	0.163	0.234	0.293
**(mean/sd)**	(0.034)	(0.030)	(0.033)	(0.074)	(0.058)	(0.059)	(0.053)	(0.037)	(0.034)
**Bivariate model**	0.169	0.223	0.293	0.123	0.221	0.299	0.166	0.252	0.320
**(mean/sd)**	(0.034)	(0.031)	(0.033)	(0.075)	(0.060)	(0.062)	(0.054)	(0.038)	(0.038)
**Improvement**	0.002	0.009	0.016	0.002	0.016	0.024	0.004	0.019	0.028

Reference Group Size = 3000	A1B1	A2B2	A3B3	C1D1	C2D2	C3D3	E1F1	E2F2	E3F3
**Single trait model**	0.175	0.225	0.284	0.126	0.206	0.273	0.179	0.248	0.301
**(mean/sd)**	(0.044)	(0.048)	(0.054)	(0.065)	(0.052)	(0.050)	(0.043)	(0.035)	(0.037)
**Bivariate model**	0.178	0.234	0.301	0.129	0.223	0.300	0.183	0.269	0.333
**(mean/sd)**	(0.045)	(0.049)	(0.054)	(0.066)	(0.054)	(0.053)	(0.044)	(0.035)	(0.038)
**Improvement**	0.002	0.009	0.017	0.003	0.017	0.026	0.004	0.021	0.032

Reference Group Size = 3500	A1B1	A2B2	A3B3	C1D1	C2D2	C3D3	E1F1	E2F2	E3F3
**Single trait model**	0.181	0.224	0.281	0.142	0.225	0.292	0.184	0.252	0.302
**(mean/sd)**	(0.031)	(0.031)	(0.035)	(0.063)	(0.052)	(0.051)	(0.042)	(0.029)	(0.031)
**Bivariate model**	0.183	0.234	0.300	0.145	0.243	0.321	0.188	0.274	0.337
**(mean/sd)**	(0.031)	(0.032)	(0.035)	(0.064)	(0.054)	(0.054)	(0.044)	(0.029)	(0.035)
**Improvement**	0.003	0.01	0.019	0.003	0.018	0.029	0.004	0.022	0.035

Reference Group Size = 4000	A1B1	A2B2	A3B3	C1D1	C2D2	C3D3	E1F1	E2F2	E3F3
**Single trait model**	0.201	0.242	0.296	0.149	0.231	0.297	0.192	0.260	0.307
**(mean/sd)**	(0.028)	(0.035)	(0.041)	(0.059)	(0.050)	(0.050)	(0.034)	(0.034)	(0.035)
**Bivariate model**	0.204	0.252	0.317	0.152	0.250	0.328	0.196	0.284	0.346
**(mean/sd)**	(0.028)	(0.035)	(0.042)	(0.060)	(0.052)	(0.053)	(0.035)	(0.034)	(0.040)
**Improvement**	0.003	0.01	0.021	0.003	0.019	0.031	0.004	0.024	0.039

Reference Group Size = 4500	A1B1	A2B2	A3B3	C1D1	C2D2	C3D3	E1F1	E2F2	E3F3
**Single trait model**	0.195	0.240	0.293	0.158	0.241	0.308	0.200	0.272	0.316
**(mean/sd)**	(0.028)	(0.040)	(0.049)	(0.059)	(0.048)	(0.047)	(0.029)	(0.028)	(0.028)
**Bivariate model**	0.198	0.250	0.314	0.161	0.262	0.343	0.204	0.296	0.357
**(mean/sd)**	(0.029)	(0.040)	(0.049)	(0.060)	(0.049)	(0.049)	(0.029)	(0.028)	(0.033)
**Improvement**	0.003	0.01	0.021	0.003	0.021	0.035	0.004	0.024	0.041
