# Supplementary figures and images for: Comparative Study of Single-Trait and Multi-Trait Genomic Prediction Models
Source: Animals (Basel). 2024 Oct 14;14(20):2961. doi: 10.3390/ani14202961 (PMC11506144; doi:10.3390/ani14202961)

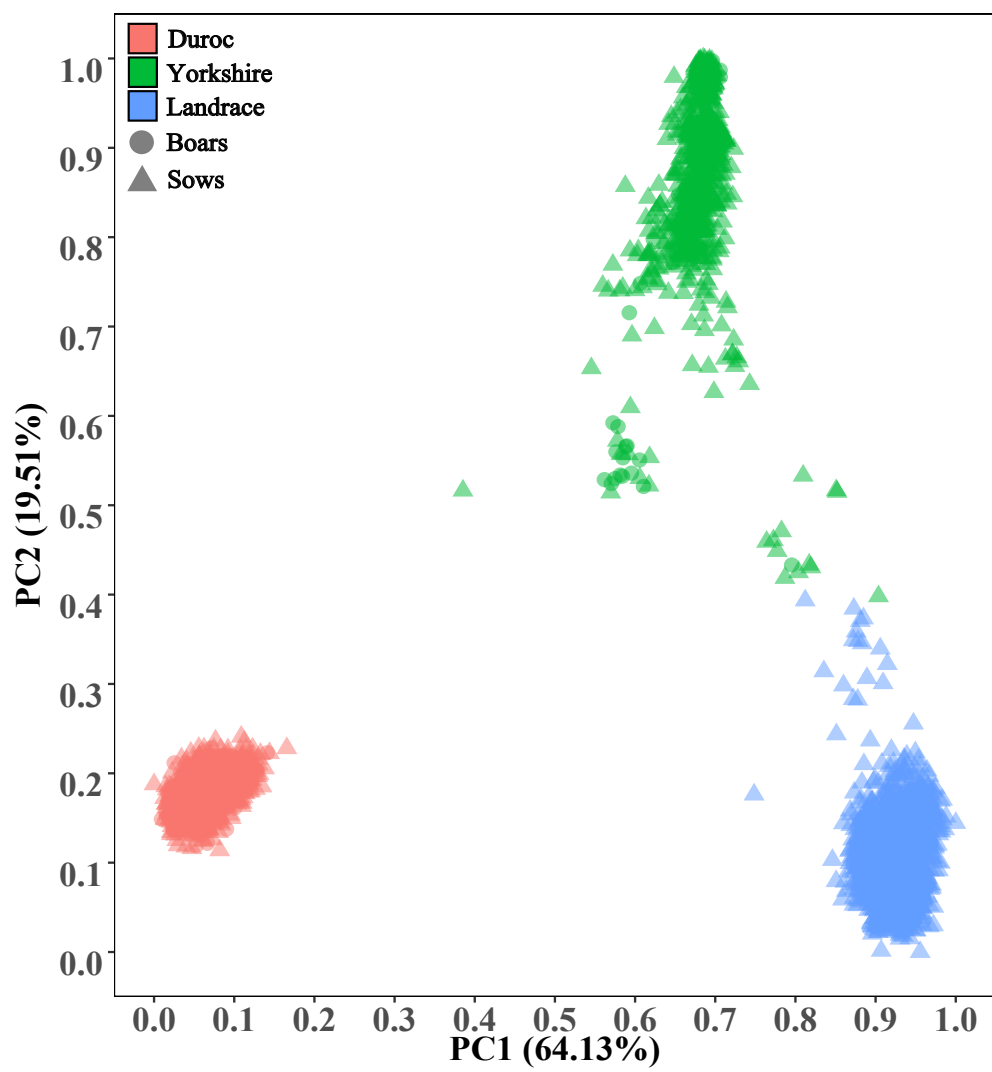

Figure S1: The distribution of principal component in the three populations.

Supplement: Supplementary file 1 [file animals-14-02961-s001.zip › Additional file 1.pdf]
